# Friends and Foes from an Ant Brain's Point of View – Neuronal Correlates of Colony Odors in a Social Insect

**DOI:** 10.1371/journal.pone.0021383

**Published:** 2011-06-23

**Authors:** Andreas Simon Brandstaetter, Wolfgang Rössler, Christoph Johannes Kleineidam

**Affiliations:** 1 Department of Behavioral Physiology and Sociobiology (Zoology II), Biozentrum, University of Würzburg, Würzburg, Germany; 2 Department of Biology, University of Konstanz, Konstanz, Germany; Claremont Colleges, United States of America

## Abstract

**Background:**

Successful cooperation depends on reliable identification of friends and foes. Social insects discriminate colony members (nestmates/friends) from foreign workers (non-nestmates/foes) by colony-specific, multi-component colony odors. Traditionally, complex processing in the brain has been regarded as crucial for colony recognition. Odor information is represented as spatial patterns of activity and processed in the primary olfactory neuropile, the antennal lobe (AL) of insects, which is analogous to the vertebrate olfactory bulb. Correlative evidence indicates that the spatial activity patterns reflect odor-quality, i.e., how an odor is perceived. For colony odors, alternatively, a sensory filter in the peripheral nervous system was suggested, causing specific anosmia to nestmate colony odors. Here, we investigate neuronal correlates of colony odors in the brain of a social insect to directly test whether they are anosmic to nestmate colony odors and whether spatial activity patterns in the AL can predict how odor qualities like “friend” and “foe” are attributed to colony odors.

**Methodology/Principal Findings:**

Using ant dummies that mimic natural conditions, we presented colony odors and investigated their neuronal representation in the ant *Camponotus floridanus*. Nestmate and non-nestmate colony odors elicited neuronal activity: In the periphery, we recorded sensory responses of olfactory receptor neurons (electroantennography), and in the brain, we measured colony odor specific spatial activity patterns in the AL (calcium imaging). Surprisingly, upon repeated stimulation with the same colony odor, spatial activity patterns were variable, and as variable as activity patterns elicited by different colony odors.

**Conclusions:**

Ants are not anosmic to nestmate colony odors. However, spatial activity patterns in the AL alone do not provide sufficient information for colony odor discrimination and this finding challenges the current notion of how odor quality is coded. Our result illustrates the enormous challenge for the nervous system to classify multi-component odors and indicates that other neuronal parameters, e.g., precise timing of neuronal activity, are likely necessary for attribution of odor quality to multi-component odors.

## Introduction

Eusocial insects live in complex societies, where the majority of individuals forego reproduction [1], [2]. Instead, the colony benefits from cooperation, and ultimately, supporting the reproduction of closely related kin results in an indirect fitness gain for colony members [3]–[5]. In order to defend common resources and reproductive relatives against rivals, it is of paramount importance for social insects to discriminate members of their own colony (nestmates) from members of foreign colonies (non-nestmates). Colony recognition in social insects is mediated by chemical cues found on the cuticle [1]. The insect cuticle is coated with a hydrophobic layer of long-chained and low-volatile hydrocarbons, originally acting as a barrier against infection and desiccation [6], [7]. In social insects, these cuticular hydrocarbons (CHC) are complex, multi-component mixtures. For a given species the components of the CHC profiles are identical, however, they differ in the ratios of components across colonies. Hence, CHC profiles are colony specific (colony odor). The chemical basis of colony recognition has been investigated most thoroughly in ants [8]–[14], yet the neuronal processes used to discriminate nestmates from non-nestmates remain elusive.

Ants detect and discriminate colony odors either by directly contacting another ant with their antennae or when antennating close-by [9], [15], [16]. The olfactory pathway of Hymenoptera is well investigated [17]–[19] and has been reviewed in great detail recently [20], [21]. Odors are received by olfactory receptor neurons (ORNs) housed in olfactory sensilla of the antenna. From there, olfactory information is relayed to functional units (glomeruli) in the first olfactory neuropile of the insect brain, the antennal lobe (AL). The insect antennal lobe is analogous to the vertebrate olfactory bulb and similar information processing mechanisms seem to act in both [22], [23]. Glomeruli are sites of synaptic interaction between ORNs, local interneurons, and output (projection) neurons. Ensembles of projection neurons relay olfactory information as a combinatorial code to higher integration centers of the insect brain (mushroom bodies and lateral horn). Since odors activate specific subsets of ORNs, this results in an odor specific glomerular activation patterns in the AL (spatial activity patterns) [24]. Earlier studies revealed that odors, which elicit similar spatial activity patterns in the AL, are perceived similarly, i.e. a similar odor quality is attributed [25], [26]. This correlation led to the suggestion that the brain readily uses activity patterns in the AL to assess odor quality. It has never been investigated whether different colony odors are represented as distinct activity patterns in the AL, and it is not known at which level of the olfactory system the odor quality ‘nestmate’ or ‘non-nestmate’ is attributed to the neuronal representation.

Traditionally, it is assumed that colony odor is compared to a neuronal template located somewhere in the nervous system and any mismatch between colony odor and neuronal template results in aggression [9], [27], [28]. Colony odors are a variable cue and may change over time in the range of weeks and months as they are influenced by environmental factors and vary with age, reproductive status, and/or caste [29]–[38]. As a consequence, a neuronal template has to be constantly updated [39]–[42]. Different mechanisms of how a neuronal template might be realized in the nervous system have been proposed and may even act in combination with each other. According to the classic idea, an internal representation of nestmate colony odor is stored as a template in higher integration centers of the insect brain, e.g. mushroom bodies and/or lateral horn [9], [27]. Sensory information is compared to the internal representation and this eventually results in recognition. Another possible mechanism is that the neuronal representation of nestmate or non-nestmate colony odor is specifically modified along the olfactory pathway, with the specific modifications acting as a template. It has been shown that learning results in changes of the neuronal representation of odors along the olfactory pathway, e.g. in the AL [43]–[46].

Alternatively, a sensory on-off filter in the periphery of the nervous system has been suggested to act as a template. Ozaki et al. [47] described an olfactory sensillum on the antenna of the ant *Camponotus japonicus* which only responded to non-nestmate, but not to nestmate colony odor. The authors suggested that the ORNs are “desensitized” to nestmates, e.g. by sensory adaptation to the constantly present nestmate colony odor. Hence, only non-nestmate specific information is relayed to the central nervous system (sensory filter), while ants are specifically anosmic to nestmate colony odor. This hypothesis is appealing due to its simplicity and it had a profound impact on the research field of colony recognition as it fundamentally challenges our current notion of how social insects identify nestmates and non-nestmates, namely by attributing the meaning ‘friend’ or ‘foe’ to a neuronal representation in the brain. However, the hypothesis of a template in form of a sensory filter fails to explain how social insects can discriminate between members of different castes and life stages within their colony under conditions in which nestmates were not detected [10], [37], [48]–[50]. Therefore, it is important to scrutinize the general validity of the suggested sensory filter hypothesis.

In a first step to understand how odor quality of colony odors is coded and how a neuronal template might be realized in the nervous system, we investigated the neuronal representation of colony odors at two levels of the olfactory system in the Florida carpenter ant *Camponotus floridanus* using a recently developed stimulation technique [51]. In a behavioral assay, we first confirmed that nestmate and non-nestmate colony odors were discriminated by workers when presented via this new stimulation technique. Then, we measured neuronal responses of ORNs of the antenna to nestmate and non-nestmate colony odors by electroantennography. Last, we used calcium imaging to monitor spatial activity patterns of projection neurons of the AL and analyzed, whether different colony odors elicit distinct activity patterns. Our results show that both nestmate and non-nestmate colony odor elicit spatial activity patterns in the AL. However, these spatial activity patterns alone are not sufficient for discrimination of nestmate and non-nestmate colony odor. Finally, we discuss which neuronal parameters of the combinatorial code of projection neurons are possibly used for quality coding of complex colony odors.

## Results

### Behavioral assay

**Figure 1:**
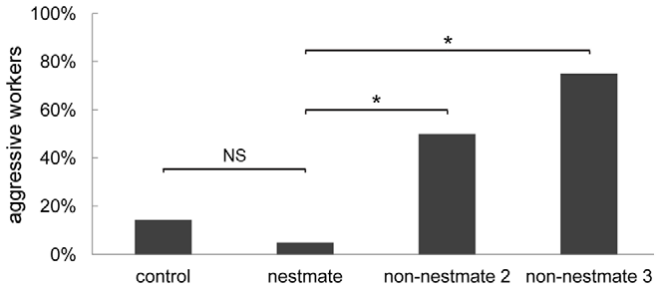
Behavioral assay. The behavioral response of workers was tested upon stimulation with a moderately heated dummy loaded with colony odor from nestmates (n = 20), non-nestmates (2) of a different population as nestmate (n = 18), non-nestmates (3) of a different species (n = 8), or solvent only (control; n = 14). Mandibular threat was counted as aggressive behavior. Significantly more workers showed aggressive behavior towards dummies loaded with non-nestmate colony odors than with nestmate colony odor (asterisks), as revealed by Benjamini-Hochberg corrected Fisher's exact tests (see results for p-values). No significant difference between nestmate colony odor loaded dummies and control was found (NS).

In a behavioral assay we tested, whether workers discriminated nestmate and non-nestmate colony odors presented via the stimulation technique used for the neurophysiological experiments in order to assure that our stimulus delivery was functional. Heated dummies loaded with NM, nNM2, nNM3 and control (dummy-delivered stimulation; see Table 1 for abbreviations) [51] were presented to tethered workers in a double-blind manner. The behavioral responses of 60 workers in total were recorded and mandibular threat was counted as aggressive behavior [15], [52], [53]. Significantly more workers responded aggressively towards dummies loaded with nNM2 and nNM3 than towards those loaded with NM, whereas no significant difference in aggressive behavior was found in response to NM and control (Figure 1; one-sided Fisher's exact test with Benjamini-Hochberg corrected p-values; nestmate vs. non-nestmate 2: p = 0.0063; nestmate vs. non-nestmate 3: p = 0.0177; nestmate vs. control: p = 0.3650). Thus, workers discriminate between heated dummies loaded with nestmate and non-nestmate colony odors without the need for tactile interaction. They show significantly more often aggressive behavior towards non-nestmate loaded dummies. Furthermore, aggressive responses to nestmate colony odor loaded dummies were rare (1 of 20) and, hence, false rejection rate of nestmate colony odor was very low.

**Table 1:** Abbreviations of colony odor stimuli presented on heated dummies.

Abbr.	colony odor extracts from
NM	nestmates, collected from the same colony
nNM1	non-nestmates of the same population as nestmates
nNM2	non-nestmates of a different population as nestmates
nNM3	non-nestmates of a different species (*C. rufipes*)
control	solvent only, no extract

### Electroantennography

**Figure 2:**
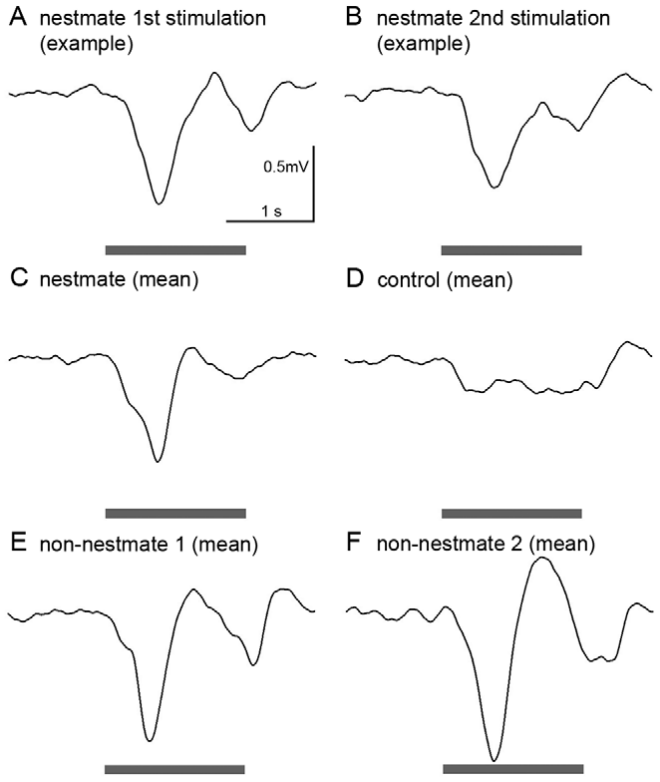
Electroantennography. **A&B:** Comparable neuronal responses of olfactory receptor neurons were measured upon repeated stimulation with the same colony odor. An example of 2 consecutive stimulations with nestmate colony odor is shown. **C–F:** Mean voltage responses (amplitude: ∼0.6–0.9 mV) of 3–8 different antennal preparations were measured upon stimulation with colony odor from nestmates (C; N = 8), non-nestmates from the same population as nestmates (E; non-nestmate 1; N = 5), and non-nestmates from a different population (F; non-nestmate 2; N = 3). Presentation of a solvent-loaded and heated dummy (D; control; N = 8) resulted in a comparably weak voltage response, probably induced by the solvent, and/or the increased temperature of the dummy. A grey bar indicates the stimulation period of 1.6 s.

We used electroantennography (EAG) as a simple neurophysiological technique to test whether ORNs of the antenna respond to colony odors of nestmates and non-nestmates. For stimulation, we used heated dummies loaded with NM, nNM1, nNM2, and control (see Table 1 for abbreviations). EAG revealed pronounced responses to colony odors in 8 antennal preparations. Repeated stimulation with the same colony odor resulted in comparable voltage responses (Figure 2 A&B). For visualization, mean response curves of the first sensory response to each odor in the tested antennae were calculated (Figure 2 C–F). NM, nNM1, and nNM2 elicited voltage responses with signal amplitudes in the range of 0.6 to 0.9 mV. In contrast, control stimulation resulted in considerably weaker signal amplitude of around 0.2 mV, which might have been induced by solvent residues and/or an increased temperature at the antennae caused by the heated dummy. The results demonstrate that dummy-delivered stimulation with both nestmate and non-nestmate colony odors evoked EAG amplitudes in a similar range, while no such responses were elicited upon control stimulation.

### Calcium imaging

Calcium imaging allows monitoring of neuronal activity by measuring changes in intracellular calcium levels using fluorescent calcium indicators, a technique that has been repeatedly used in ants [19], [22], [51], [54], [55]. As a test stimulus for functionality, we presented a general odor delivered via an air-stream (air-delivered octanol at a dilution of 10^−1^) and measured neuronal activity in 22 animals. For colony odor stimulation we used NM, nNM1, nNM2, nNM3 and control (see Table 1 for abbreviations). In 8 preparations all odors were tested at least twice.

**Figure 3:**
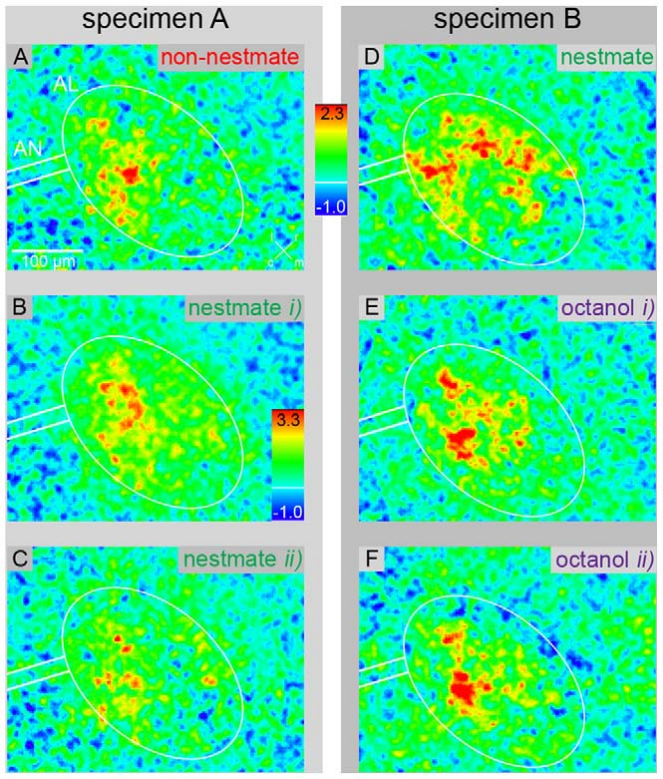
False-color coded neuronal activity (calcium imaging) in the antennal lobe (AL), in response to different odors. Examples of 2 different individuals (specimen A and B). Dummy-delivered stimulation with non-nestmate (A; different population as nestmate) and nestmate colony odor (C; NM) resulted in neuronal activity within the same region of the AL and in a similar range of intensities. Neuronal activity induced by NM was highly variable across animals (cp. C&D). Air-delivered octanol stimulation resulted in activity patterns that clearly differ from NM responses (cp. D&E). Repeated stimulation with octanol resulted in a consistent neuronal representation (cp. E&F), whereas spatial activity patterns and response intensity upon repeated NM stimulation were variable (cp. B&C). Time period between repeated stimulations was at least 24 min. Red indicates areas of high neuronal activity and a colored bar denotes the fluorescence change [ΔF/F]. To visualize the spatial activity pattern, intensity range of B is individually scaled as indicated by the individual scale bar.

NM and the three different non-nestmate colony odors (nNM) elicited neuronal activity in the AL with response intensities in a similar range (Figure 3 A&C). No response was measured upon control stimulation (Figure S1). Across animals, colony odor stimulation showed highly variable neuronal activity patterns (Figure 3 C&D). This variability can be expected as colony odors change over time [36], [40], [41], and measurements were performed over the course of several months. Furthermore, activity patterns cannot be easily compared across individuals, as the AL of *C. floridanus* comprises ∼450 small and densely-packed glomeruli [19] and, hence, calcium signals cannot be assigned to individual identified glomeruli. Therefore, in the following analyses neuronal activity patterns in response to different colony odors were compared exclusively within animals.

Within individual ants, NM and nNM activated similar AL regions (Figure 3 A&C), i.e. spatial activity patterns were largely overlapping. In contrast, the spatial activity patterns in response to air-delivered octanol differed considerably from activity patterns elicited by colony odors (cp. Figure 3 D&E). Repeated stimulation with octanol resulted in consistent activity patterns (Figure 3 E&F), as shown earlier in another study [19], whereas repeated stimulation with colony odor resulted in surprisingly variable neuronal responses in terms of intensity ranges and activity patterns (Figure 3 B&C). Octanol and colony odor were presented with different stimulation techniques (air- and dummy-delivered stimulation, respectively), and therefore we did not analyze octanol elicited activity patterns any further. It is important to note, though, that dummy-delivered stimulation with a single-component odor (nerolic acid) elicited stable activity patterns in an earlier study [51], and hence, the variability in activity patterns we measured in response to colony odors cannot be simply attributed to the stimulation technique we used.

In order to quantify variability between neuronal representations of NM and nNM, we performed a correlation analysis. The global intensity level of neuronal responses is not taken into account in a correlation analysis, and this allowed us to directly compare the spatial activity patterns elicited by different colony odors. We reduced the spatial resolution of the calcium image stacks to reduce noise level. Low-resolution activity patterns in response to NM and nNM looked very similar, but activity patterns still depicted distinct differences between activity patterns of e.g. NM and octanol (Figure S2). Within animals, we calculated the coefficients of correlation over time by pair-wise comparing i) neuronal responses upon repeated stimulation with the same odor (equal odor pairs) and ii) responses upon NM stimulation to responses upon stimulation with another odor (unequal odor pairs).

**Figure 4:**
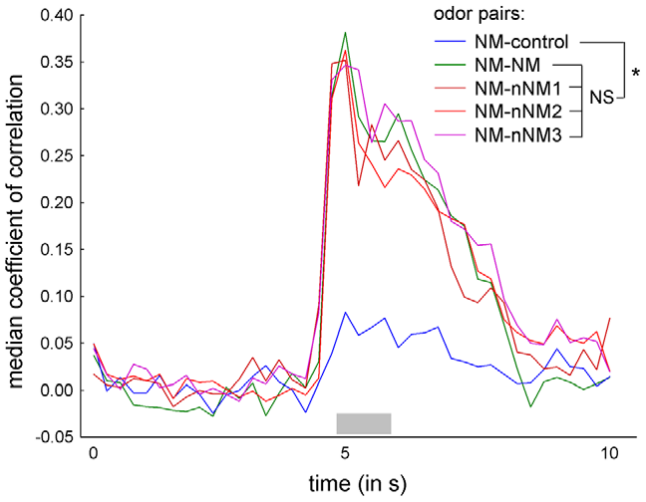
Correlation analysis of neuronal responses to different colony odors. In order to compare the variability in activity patterns elicited by different colony odors, coefficients of correlation were calculated comparing repeated stimulation with nestmate colony odor (NM-NM; see Table 1 for abbreviations), stimulation with nestmate to different non-nestmate colony odors (NM-nNM1/2/3), and nestmate colony odor to control stimulation (NM-control) within 8 animals. Prior to stimulation, coefficients of correlation are close to 0 for all odor pairs. Upon stimulation (a grey bar indicates the stimulation period of 1 s), coefficients of correlation increase considerably for NM-NM and NM-nNM1/2/3, whereas they remain low for NM-control. After stimulation, coefficients of correlation return to baseline. A Friedman test revealed a significant difference in the coefficients of correlation during stimulation. Post-hoc tests showed that the coefficients of correlation for colony odor pairs are not significantly different (NS), whereas a significant difference was found between NM-control and all colony odor pairs (asterisk; Benjamini-Hochberg corrected Wilcoxon-matched-pairs tests; see Table S2 for p-values).

For visualization, we pooled the coefficients of correlation of corresponding odor pairs of all 8 animals by calculating the median and plotted those of NM-NM and unequal odor pairs (Figure 4). Prior to stimulation, correlation was close to 0. During stimulus presentation, correlation increased considerably for NM-NM and NM-nNM1/2/3, and decreased back to baseline after the end of stimulation. For NM-control, correlation remained low during the whole recording. Coefficients of correlation of equal odor pairs (repeated stimulation with the same odor) were all in the same range upon stimulation (Table S1). To test whether the plotted coefficients of correlation for NM-NM and unequal odor pairs differed significantly during the stimulation period, we used a Friedman test and found a significant difference (Friedman rank sum test; chi^2^ = 16.6, DF = 4, p = 0.0023). As a post-hoc test, we compared the odor pairs using Wilcoxon-matched-pairs tests and corrected the p-values for multiple testing according to the Benjamini-Hochberg method (Table S2). Whereas all colony odor pairs where significantly different from the NM-control odor pair, no significant different between colony odor pairs was found.

In summary, we find that the correlation of activity patterns elicited by repeated NM stimulation was not significantly different from the correlation of activity patterns elicited by stimulation with different colony odors (i.e. unequal colony odor pairs: NM-nNM1/2/3). Based on our correlation analysis, we conclude that on a large scale colony odors elicit similar spatial activity patterns in the AL. Within this large scale of colony odor representations, both, the activity patterns for nestmate and non-nestmate colony odor are variable to a similar extent. Thus, the spatial representation of nestmate- and non-nestmate is not specific enough to provide the nervous system with sufficient information for discrimination.

## Discussion

In this study, we measured neuronal correlates of colony odors at two levels of the olfactory system of the carpenter ant *Camponotus floridanus*. Our results provide neurophysiological evidence that ants can perceive colony odors from both, nestmates and non-nestmates, contradicting the sensory filter hypothesis for colony recognition. At the level of the antennal lobe (AL; projection neurons) spatial activity patterns in response to colony odors were variable – even upon repeated stimulation with the same colony odor – and we did not find any significant differences in activity patterns upon stimulation with different colony odors. Thus, spatial activity patterns alone are not sufficient to classify colony odors as nestmate or non-nestmate specific. Nevertheless, behavioral experiments presented here and in earlier studies show that the nervous system is well able to classify nestmate and non-nestmate colony odors [15], [53], [56]–[59], despite the variable neuronal representation of complex, multi-component odors that we found in this study. Our results raise the question which parameters of neuronal activity are used besides spatial activity patterns to assess odor quality.

Both, electroantennography and calcium imaging, revealed neuronal activity in response to stimulation with nestmate colony odor in olfactory receptor neurons (ORNs) of the antenna and in projection neurons of the AL. There were no pronounced differences in the summed voltage responses of ORNs and in the spatial activity patterns in the AL elicited by nestmate and non-nestmate colony odor. This finding clearly contradicts the model proposed by Ozaki et al. on the closely related ant species *C. japonicus* [47] that complete adaptation to the nestmate specific ratios of cuticular hydrocarbons blocks perception of nestmate odor at the level of the antennal sensilla (nestmate specific anosmia). As the olfactory system in both *Camponotus* species is similarly organized [18], [19], we conclude that a neuronal template for colony recognition is extremely unlikely to be implemented in form of a sensory on-off filter at the level of ORNs in the antenna of ants. Our conclusion is also supported by other studies, which consistently showed that template reformation, i.e. the process of updating the neuronal template to a changing colony odor is a relatively slow process, taking several hours [53], [60]. This is much longer than the time period expected for sensory adaptation at antennal ORN level.

What causes the high variability of spatial activity patterns within individuals as measured in response to repeated stimulation with the same colony odor? We obtained colony odors from extracts of postpharyngeal glands, which contain the same components at equivalent ratios as the CHC profile [8], [61], [62]. These extracts were readily discriminated by ants even without physical contact to the extract-loaded dummies [15]. Compared to an earlier study [51], we improved stimulus application by moderately heating the dummies to increase colony odor concentration in the headspace of dummies. Recently, a number of temperature-sensitive glomeruli have been reported for the dorsal region of the AL in leaf-cutting ants [63]. However, we did not measure any unspecific temperature responses, probably because we were investigating the anterior part of the AL.

In a behavioral assay, we assured that our dummy-delivered stimulation for presentation of colony odors is functional and that an increased temperature of the dummies does not alter the quality of the colony odor stimuli. The significantly different behavioral responses to stimulation with nestmate and non-nestmate colony odor and the low rate of false rejection of nestmate colony odor show that ants can well discriminate the different stimuli and, hence, dummy-delivered stimulation can be considered functional and well-suited for presentation of low-volatile colony odors.

We could show that dummy-delivered stimulation with multi-component colony odors resulted in variable neuronal responses within animals, however, an earlier study showed that dummy-delivered stimulation with a single component, namely nerolic acid, the releaser component of *C. floridanus*' trail pheromone, resulted in stable spatial activity patterns across individuals and trials [51]. The same was true for air-delivered stimulation with nerolic acid [19]. We conclude that the variable neuronal responses to colony odors cannot originate from our dummy-delivered stimulation *per se*.

Individual components of colony odors have different chemo-physical properties. Depending on their vapor pressure, temperature, and humidity they evaporate into headspace at different rates. Thus, the multi-component odor stimulus arriving at the antenna of an ant not only depends on the chemical composition of the colony odor, but may also vary depending on external physical factors like temperature, humidity as well as the distance and diffusion rate between colony odor source and receiver. For presentation of colony odors, we used a stimulation technique resembling the natural situation by simulating close-range colony odor detection from a nearby nestmate or non-nestmate. Experimental conditions were kept as constant as possible, yet even minute differences in external factors may subtly influence the composition of the low-volatile, multi-component colony odor stimulus, resulting in stimulus variability. A recent study in moth showed that the ratios of odor components can vary to some degree without changing the odor's behavioral significance [64].

Olfactory information is integrated and processed in the AL network by interactions between glomeruli [24], [65]. Detection and discrimination of complex, multi-component odors require extensive neuronal processing, and even small variations between odor stimuli arriving at the antenna may impact the resulting spatial activity patterns [64], [66]. We hypothesize that due to the different chemo-physical properties of the various colony odor components, stimulus variability is accentuated through odor information processing by the antennal lobe network, and this leads to the measured variability in spatial activity patterns. It has to be noted that our behavioral assay confirmed: The variability in spatial activity patterns does not prevent workers from discriminating nestmate and non-nestmate colony odors. In order to allow accurate colony recognition, the nervous system needs to classify colony odors as nestmate and non-nestmate specific despite their variable neuronal representation.

Which parameters are used by the nervous system to classify colony odors? It has been shown that spatial activity patterns highly correlate with perceived odor quality [25], [26]. However, here we show that different colony odors activated largely overlapping AL areas. Overlapping and equally variable spatial activity patterns for different colony odors may be expected, given that the chemical profiles of nestmate and non-nestmate colony odor contain the same chemical components, only at differing ratios. Interestingly, spatial activity patterns upon stimulation with colony odor of another *Camponotus* species (*C. rufipes*) were also not significantly different from activity patterns elicited by colony odors of *C. floridanus*. Both, *C. rufipes* and *C. floridanus*' colony odors probably contain linear and methyl-branched alkanes within the same range of chain length, and a large overlap of chemical profiles would explain the similarity of neuronal responses elicited by *C. floridanus* and *C. rufipes* colony odors. Recently, it has been shown that workers of a sub-colony in which the colony odor was supplemented with only a single component (a di-methylated hydrocarbon) are discriminated and attacked by workers of a matching but unmanipulated sub-colony [28]. Based on our measurements of neuronal responses to colony odors of *C. floridanus* and *C. rufipes*, we expect only little impact of a single component on the spatial activity patterns in the AL. We suggest that the overlapping spatial activity patterns code for general odor quality like ‘colony odor’. Because of the variability of spatial activity patterns in response to colony odors, either many patterns have to be learned in order to discriminate nestmates from non-nestmates or other parameters besides the spatial activity pattern are used for colony odor classification. Several studies emphasize the importance of precise timing of neuronal activity for discrimination of chemically similar odors and odor blends [26], [67]–[71]. The complex interplay between glomeruli via local interneurons results in distinct temporal firing patterns of projection neurons of the AL, which may be specifically modified (e.g. as a result of template reformation, i.e. learning). The importance of the AL in providing mechanisms for colony recognition has been demonstrated for another ant species (*C. aethiops*) [60], and in particular projection neurons from the AL possibly accommodate a memory trace [72]. Specific colony odors may result in synchronous activity in ensembles of projection neurons leading to patterns of coincidence in postsynaptic neurons at the next levels of the olfactory pathway, i.e. the mushroom bodies or the lateral horn. Thus, temporal activity patterns of AL projection neurons may suffice to code for nestmate or non-nestmate specificity. Furthermore, distinct spatio-temporal activity patterns in higher integration centers of the insect brain (e.g. Kenyon cells in the mushroom bodies) may be compared to a template stored in long-term memory, which then results in recognition. Memory consolidation is accompanied by a calcium induced long-term structural rearrangement of mushroom body synapses [73], [74] and this may be important for template reformation.

As our present study clearly shows that ants are not anosmic to nestmate colony odors and that information about different colony odors are transferred equally to olfactory centers in the brain, the future challenge is to unveil what kind of information is used to classify nestmate and non-nestmate colony odors, and in general, how insects assess the quality of multi-component odors. Multi-component odors constitute highly complex stimuli, and most probably animals are generally faced with the problem that these may elicit variable neuronal responses which have to be classified correctly by the nervous system to allow accurate odor recognition. Colony recognition in social insects is an excellent model system to study the coding of odor quality and long-term memory mechanisms underlying recognition of complex, multi-component odors, as it allows investigating the neuronal representation of the same odor stimulus with potentially opposing attributes: friend or foe.

## Materials and Methods

### Ethics Statement

The performed experiments comply with the current laws of the Federal Republic of Germany and collection of founding queens for laboratory colonies conformed to the laws of the United States of America and the Oriental Republic of Uruguay effective at time of collection.

### Animals

*C. floridanus* is an evolutionary-derived eusocial species with colonies consisting of more than 10,000 individuals but only one single-mated queen [75]. Genetic homogeneity within colonies is high and heritable components of the colony odor are probably important for colony recognition in this species [57], [58]. Workers show distinct colony recognition behavior, which has been studied in great detail [56]–[59]. Their cuticular hydrocarbon profiles mainly consist of linear and methyl-branched alkanes of chain lengths between C29 and C32 [32], [76].

Experimental colonies were raised from founding queens collected by A. Endler and S. Diedering in Florida (USA) at Florida Keys after mating flight. Colonies were kept in the laboratory in artificial plaster nests at a constant temperature of 25°C and 50% humidity (12 h/12 h photoperiod) and provided with artificial diet [77], honey-water, and dead cockroaches (*Nauphoeta cinerea*) twice a week and water ad libitum. Colony size was approximately 4000 ants. Behavioral experiments were conducted with workers from a colony with a founding queen collected at Sugarloaf Shores in July 2003. Neurophysiological experiments were conducted with large workers (head width >3 mm) and nestmate colony odor was obtained from small workers (head width <3 mm) of the same colony (NM). Non-nestmate colony odors were obtained from small workers, whose founding queens had been collected at Sugarloaf Shores in July 2002 and 2003 (same population as nestmates; nNM1), and Orchid Island in August 2001 (different population than nestmates; nNM2), respectively. Non-nestmate colony odor of a different species was obtained from small workers of a *Camponotus rufipes* colony, with a founding queen collected in La Pedreras (Uruguay) by O. Geissler in December 2002 (nNM3). Rearing conditions were identical to those of *C. floridanus* colonies. Abbreviations for colony odor stimuli are described in Table 1.

### Colony odor extraction

Colony odors were obtained from postpharyngeal glands (PPG), which contain the same components as the colony odor found on the cuticle in equivalent ratios [8], [61], [62]. Using PPG extracts for stimulation is advantageous in comparison to stimulation with alive or freshly killed ants, where results may be confounded by pheromone release due to stress during the experiment or the sacrificing process, respectively. Furthermore, PPG extracts contain remarkably less short-chain components, which do not belong to the hydrocarbons constituting the colony odor, than hexane cuticle washes [15]. PPGs were dissected and extracted in hexane for at least 2 h before loading them onto dummies as described in detail previously [15]. As colony odors change over time in the range of weeks and months [36], [40], [41], we used only PPG extracts, which had been prepared maximally 5 days in advance.

### Stimulus delivery

For stimulation with colony odors, we used a recently developed stimulus delivery technique, which closely mimics the natural situation of odor dispersal from solid surfaces like e.g. an insect cuticle: a dummy is loaded with an odor and moved into close vicinity of the antenna. This has been shown to be advantageous for stimulation with low-volatile odors [51]. In order to further increase colony odor concentration in headspace, dummies were heated to a temperature of 40°C before applying the colony odor (behavioral assay and EAG: KTY temperature sensor heated by a constant current power source, Conrad Electronic SE; calcium imaging: Firerod Cartridge Heater operated by a F4SL ramping temperature controller, Watlow GmbH). Prior to stimulation, hexane-rinsed dummies were loaded with 20 µl of colony odor using hexane-rinsed Hamilton syringes (Hamilton Company), and the solvent was allowed to evaporate for 2 min. Room temperature was kept constant at 25°C.

In the behavioral assay, one colony odor was presented per animal in a double-blind manner. For EAG recordings, a colony odor was presented 2 to 3 times with an inter-stimulus-interval of ∼1 min. Only the first EAG response was used for further analysis. Subsequently, a different colony odor was presented. The overall sequence of colony odors was pseudo-random. For calcium imaging, colony odors were presented in a fixed sequence with an inter-stimulus-interval of 4 min as follows: nNM2 – NM – control – nNM1 – nNM3 – control. Again, this stimulation sequence was repeated 2 to 3 times, and the inter-stimulus-interval between repeated stimulation with the same colony odor was at least 24 min.

### Behavioral assay

Workers were immobilized on ice and a minutien pin was attached to the thorax, using gently heated wax. A small Styrofoam ball (diameter = 1 cm) was offered each tethered worker, which it willingly grabbed and started walking on it. The worker was shielded with a metal box and an acrylic glass front to minimize disturbance by air currents. Right in front of the tethered worker, a small hole in the acrylic glass allowed stimulus presentation. After an accommodation phase of 5 min, the heated dummy loaded with NM, nNM2, nNM3, or control was presented in a double-blind manner without allowing tactile interaction, and the behavioral response within a time frame of 10 s was recorded. Mandibular threat was counted as aggressive behavior. All experiments were conducted at red light conditions to exclude any visual cues. For statistical analysis, we performed one-sided Fisher's exact tests and corrected the p-values for multiple testing according to the Benjamini-Hochberg method [78].

### Electroantennography

A cut antenna of a worker was mounted between 2 chlorinated silver electrodes and the sum potential of ORNs in response to NM, nNM1, and nNM2 during a stimulation period of 1.6 s was measured. For visualization, mean response curves of the first sensory response to each odor were calculated for 8 antennae. Note that not all odors could be tested in all of the 8 antennal preparations. Details on the experimental setup and data processing have been described earlier [51].

### Calcium imaging and data evaluation

Projection neurons of the AL were retrogradely loaded with Fura2-dextran (potassium salt, 10 000 MW, F3029, Molecular Probes), and ratio-metric recordings at 340 and 380 nm excitation wavelength were obtained at a frame rate of 4 Hz as detailed previously [51]. We prepared 172 workers of which 82 (47.7%) showed bright staining of projection neurons in the AL. Dummy-delivered stimulation with NM, nNM1, nNM2, and nNM3 started 5 s after start of recording for a stimulation period of 1 s.

Imaging data were analyzed using custom software written in Interactive Data Language (IDL 6.0; ITT Visual Information Solutions, Boulder, CO, USA) by Giovanni Galizia and Mathias Ditzen (University of Konstanz, Germany). We calculated the ratio of fluorescence intensity of the images taken at 340 and 380 nm excitation wavelength for each pair as: R = F_340_/F_380_ and corrected manually for possible movement of the AL between measurements. To visualize neuronal responses to the different colony odors as false-color coded images, we subtracted the average of 3 frames prior to stimulation from the average of 3 frames during stimulation.

In order to quantify variability in neuronal responses to different colony odors, we compared neuronal activity patterns using a pixel-based Pearson's product-moment correlation analysis over time (MS Office Excel 2007 SP2). We reduced noise by reducing the spatial resolution of image stacks by a factor of 8. This resulted in a pixel size of 20×20 µm, which approximately corresponds to the size of one glomerulus in *C. floridanus* and suffices to discriminate distinct spatial activity patterns (cp. Figure S2 E&F). To compensate for different onset of neuronal responses, we calculated the coefficients of correlation for a floating time window of 4 frames (1 s), which moved frame-by-frame through the whole recording time of 40 frames (10 s). Because of the high number of glomeruli in the AL of *C. floridanus* [19], calcium signals could not be assigned to identified glomeruli, as it is possible e.g. in *Apis mellifera* [79]. For this reason, neuronal activation patterns were only compared within individual animals. Pearson's coefficient of correlation was calculated pairwise, i) for equal odor pairs, i.e. for repeated stimulation with the same odor, comparing 1^st^ stimulation with odor A to 2^nd^ stimulation with odor A (A1–A2) and ii) for unequal odor pairs, i.e. for stimulation with two different odors (see Table 1 for abbreviations). In order to correct for possible effects of stimulation sequence, we calculated 2 coefficients of correlation for unequal odor pairs, comparing 1^st^ stimulation with odor A to 2^nd^ stimulation with odor B and vice versa (A1–B2 and A2–B1), and used their median for further analysis. For repeated odor stimulations within each individual, we calculated the median of the coefficients of correlation for all possible odor pairs, and used these medians for further analysis. Only for visualization, coefficients of correlation for NM-NM and unequal odor pairs of all 8 animals were pooled (by calculating median curves) and plotted (Statistica 9.1, Statsoft).

We tested for significant differences in coefficients of correlation of the equal odor pair NM-NM and unequal odor pairs within individual animals during stimulus presentation using a Friedman test (R statistic software 2.10.1, The R Foundation for Statistical Computing). As a post-hoc test to identify which odor pairs were significantly different from each other, we performed Wilcoxon-matched-pairs tests. To correct for multiple testing, we adjusted the, p-values according to the Benjamini-Hochberg method [78].

## Supporting Information

Figure S1 **False-color coded neuronal activity (calcium imaging) in response to control stimulation.** Presentation of a heated dummy loaded with solvent only did not result in changes of neuronal activity within the AL.
(TIF)

Figure S2 **Low-resolution, false-color coded images of neuronal activity (calcium imaging) in the AL of 2 individuals (specimen A&B, see** **Figure 3** **).** For the correlation analysis, spatial resolution of the recorded image stacks was reduced to reduce noise and trimmed to an area corresponding to the AL. Spatial activity patterns in response to colony odors appear similar (A–D), whereas the pattern in response to octanol is different from that to nestmate colony odor (E&F; intensity ranges are individually scaled for visualization). Nestmate and non-nestmate 1/2/3 correspond to the abbreviations described in Table 1 (NM and nNM1/2/3, respectively).
(TIF)

Table S1 **Coefficients of correlation of neuronal responses to colony odors.**
(DOC)

Table S2 **Correlation analysis: p-values of post-hoc Wilcoxon-matched-pairs tests.**
(DOC)
