# Supplementary figures and images for: Friends and Foes from an Ant Brain's Point of View – Neuronal Correlates of Colony Odors in a Social Insect
Source: PLoS One. 2011 Jun 23;6(6):e21383. doi: 10.1371/journal.pone.0021383 (PMC3121771; doi:10.1371/journal.pone.0021383)

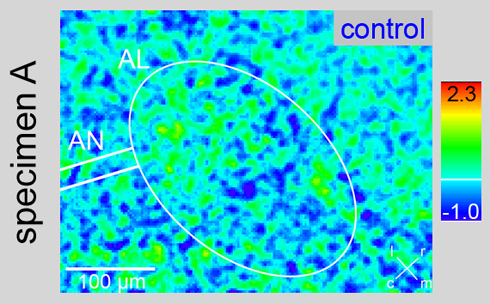

Supplement: Figure S1 — False-color coded neuronal activity (calcium imaging) in response to control stimulation. Presentation of a heated dummy loaded with solvent only did not result in changes of neuronal activity within the AL. (TIF) [file pone.0021383.s001.tif]

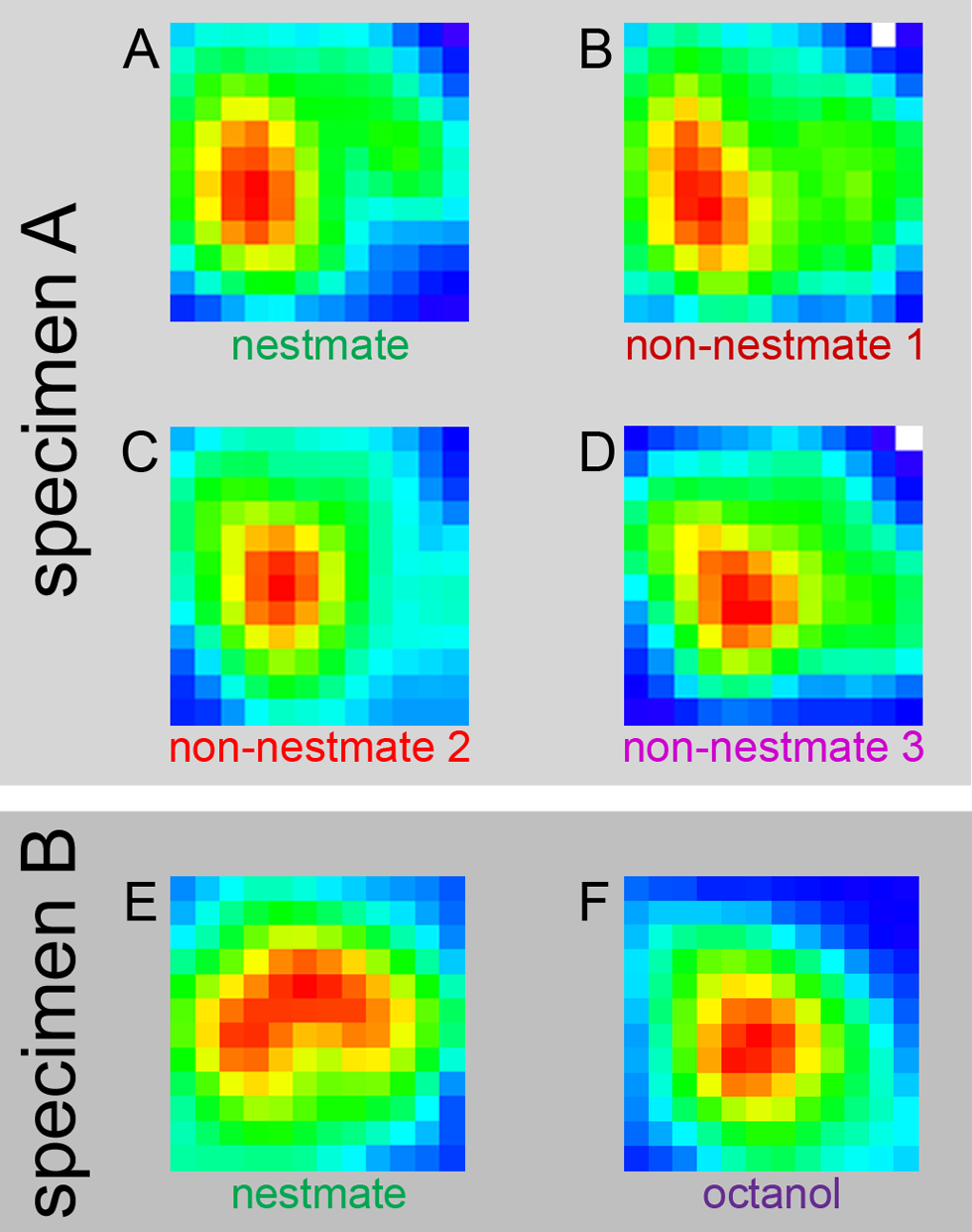

Supplement: Figure S2 — Low-resolution, false-color coded images of neuronal activity (calcium imaging) in the AL of 2 individuals (specimen A&B, see Figure 3 ). For the correlation analysis, spatial resolution of the recorded image stacks was reduced to reduce noise and trimmed to an area corresponding to the AL. Spatial activity patterns in response to colony odors appear similar (A–D), whereas the pattern in response to octanol is different from that to nestmate colony odor (E&F; intensity ranges are individually scaled for visualization). Nestmate and non-nestmate 1/2/3 correspond to the abbreviations described in Table 1 (NM and nNM1/2/3, respectively). (TIF) [file pone.0021383.s002.tif]
