# Supplementary material for: Friends and Foes from an Ant Brain's Point of View – Neuronal Correlates of Colony Odors in a Social Insect
Source: PLoS One. 2011 Jun 23;6(6):e21383. doi: 10.1371/journal.pone.0021383 (PMC3121771; doi:10.1371/journal.pone.0021383)
Supplement: Table S1 — Coefficients of correlation of neuronal responses to colony odors. (DOC) [file pone.0021383.s003.doc]

**Table S1. Coefficients of correlation of neuronal responses to colony odors.**

| **correlated odor pair** | **coefficient of correlation** | | |
| --- | --- | --- | --- |
|  | minimum | maximum | median |
| NM-NM | 0.090 | 0.671 | 0.382 |
| nNM1-nNM1 | 0.037 | 0.468 | 0.347 |
| nNM2-nNM2 | 0.030 | 0.631 | 0.400 |
| nNM3-nNM3 | -0.038 | 0.589 | 0.297 |
| NM-nNM1 | 0.046 | 0.545 | 0.352 |
| NM-nNM2 | 0.062 | 0.454 | 0.362 |
| NM-nNM3 | -0.029 | 0.610 | 0.347 |
| NM-control | -0.030 | 0.329 | 0.083 |

Minimal, maximal, and median coefficients of correlation of odor pairs during stimulation are listed (see Tab. 1 for abbreviations). Coefficients of correlation of equal odor pairs (i.e. repeated stimulation with the same colony odor) are all in the same range.
