# Supplementary material for: Friends and Foes from an Ant Brain's Point of View – Neuronal Correlates of Colony Odors in a Social Insect
Source: PLoS One. 2011 Jun 23;6(6):e21383. doi: 10.1371/journal.pone.0021383 (PMC3121771; doi:10.1371/journal.pone.0021383)
Supplement: Table S2 — Correlation analysis: p-values of post-hoc Wilcoxon-matched-pairs tests. (DOC) [file pone.0021383.s004.doc]

**Table S2. Correlation analysis: p-values of post-hoc Wilcoxon-matched-pairs tests.** p-values where adjusted for multiple testing according to the Benjamini-Hochberg method.

| **odor pairs** | **NM-control** | **NM-NM** | **NM-nNM1** | **NM-nNM2** |
| --- | --- | --- | --- | --- |
| **NM-NM** | 0.026 | - | - | - |
| **NM-nNM1** | 0.026 | 0.391 | - | - |
| **NM-nNM2** | 0.026 | 0.768 | 0.844 | - |
| **NM-nNM3** | 0.039 | 0.844 | 0.844 | 0.844 |
